# A Fano-resonance plasmonic assembly for broadband-enhanced coherent anti-Stokes Raman scattering

**DOI:** 10.1038/s41598-023-33894-6

**Published:** 2023-05-04

**Authors:** Yujia Zhang, Minjian Lu, Zhendong Zhu, Yan Li, Haoyun Wei

**Affiliations:** 1 State Key Laboratory of Precision Measurement Technology & Instruments, Department of Precision Instrument, Tsinghua University, Beijing, 100084 China; 2 National Institute of Metrology, Beijing, 100029 China

**Keywords:** Metamaterials, Metamaterials

## Abstract

Surface-enhanced coherent anti-Stokes Raman scattering (SECARS) technique has triggered huge interests due to the significant signal enhancement for high-sensitivity detection. Previous SECARS work has tended to focus only on the enhancement effect at a certain combination of frequencies, more suitable for single-frequency CARS. In this work, based on the enhancement factor for broadband SECARS excitation process, a novel Fano resonance plasmonic nanostructure for SECARS is studied. In addition to the 12 orders of magnitude enhancement effect that can be realized under single-frequency CARS, this structure also shows huge enhancement under broadband CARS in a wide wavenumber region, covering most of the fingerprint region. This geometrically-tunable Fano plasmonic nanostructure provides a way to realize broadband-enhanced CARS, with potentials in single-molecular monitoring and high-selectivity biochemical detection.

## Introduction

Surface enhanced Raman scattering (SERS) has been widely researched for huge Raman signal enhancement by modified plasmonic nano-structures^1,2^. The local electric field intensity can be increased by SERS technique of the order of ~ 10^3^–10^10^, which has been applied in single-molecule detection or biosensing at nanoscale^3,4^. Actually, coherent anti-Stokes Raman scattering, as a nonlinear four-wave mixing (FWM) process, shows a larger potency in achieving stronger Raman signal due to the coherent process^5,6^. Combined with the similar enhancement mechanism, the SECARS technique has been proposed. Compared with SERS process, both pump and anti-Stokes local-enhanced fields of CARS contribute more to the final Raman signals. Theoretically the enhancement factor of CARS, i.e., $G_{{{\text{SECARS}}}}$ can be around 10^8^–10^24^, which is 4–12 orders of magnitude improvement compared with SERS. However, experiments on SECARS show much lower $G_{{{\text{SECARS}}}}$ than expected. The most possible reason is that the enhancement of the pump ($g_{{\text{P}}}$), Stokes ($g_{{\text{S}}}$) and anti-Stokes ($g_{{{\text{AS}}}}$) light cannot meet the same spatial position, and thus the EF of individual wavelength may not be large enough for total EF^6^.

In order to realize the same “hot spot” position of the exciting light and anti-Stokes light, Fano-resonance structures are proposed and show huge potency in SECARS technique^7,8^. The Fano resonance is realized by the destructive interference between a broad bright mode and a narrow dark mode, generating a special asymmetric lineshape of the absorption profile^9–11^. When the wavelengths of exciting light and anti-Stokes light match with the Fano dips or peaks, $g_{{\text{P}}}$, $g_{{\text{S}}}$ and $g_{{{\text{AS}}}}$ simultaneously reach to a large magnitude at the same “hot spot” position leading to a relatively large $G_{{{\text{SECARS}}}}$^6^. What is more, by varying the geometrical structure parameters, the Fano dips or peaks can be flexibly tuned to suit the resonance wavelength of CARS, which helps to obtain larger $G_{{{\text{SECARS}}}}$. Hence, huge enhancement effect and local-field distribution tunability make the Fano-resonance structure a powerful tool for SECARS, and has been applied in single-molecule sensing, significantly improving the signal sensitivity^5,12^. Although a series of novel Fano-resonance nanostructures have been designed and fabricated, the Fano plasmonic structures for the application of SECARS are relative few^5,7^.

With the development of broadband CARS technology, such as the traditional multiplex CARS, broadband SECARS technology needs to be taken into account^13–15^. Different from single-frequency SECARS, the enhancement effect of broadband SECARS also depends on the spectral bandwidth of both pump and Stokes beam. When different wavelength combinations of pump and Stokes light correspond to the same Raman wavenumber, all the enhancement effects under these combinations contribute to the final CARS intensity at this wavenumber. Designing suitable nanostructures which enable broadband CARS enhancement, is necessary for broadband Raman region sensing, achieving simultaneous enhancement of multiple chemical components^16^.

In this work, the $G_{{{\text{SECARS}}}}$ for broadband CARS is first discussed, which is derived from broadband CARS excitation process to evaluate the enhancement effect considering a large wavenumber region. A periodic metal plasmonic nanostructure whose unit consists of a medial square-shaped part and two side-coupled parallel rods, is then proposed for SECARS with Fano resonances. By changing the geometrical structure parameters, the Fano peaks/dips can be easily modified to desired frequency. And this kind of structure shows a significant enhancement in both single-frequency CARS and broadband CARS, covering the Raman region from 500 to 2000 cm^−1^, which benefits to the rich variety of chemical bonds sensing in fingerprint region.

## $G_{{{\text{SECARS}}}}$ for broadband CARS

For broadband SECARS, different frequency combinations of the pump and Stokes may contribute to the same anti-Stokes frequency leading to a more significant enhancement. Here, an alternative evaluation method of EF applicable to broadband CARS is first derived. Under the broadband CARS condition, we assume that both the pump and Stokes light are broadband spectra. The probe light is provided by the same light source as the pump light. Considering the realistic stimulation process of broadband CARS, the pump light and Stokes light first interact with each other, then the broadband probe light excites the molecular vibrations to generate the anti-Stokes light. The final anti-Stokes electric field intensity can be described as^17–19^:

1 $$\begin{aligned} & E_{AS} (\omega ) \propto f_{AS} (\omega )\int_{0}^{\infty } {A(\Omega )} \chi^{(3)} (\Omega ) \cdot (f_{probe} (\omega - \Omega ) \cdot E_{probe} (\omega - \Omega ))d\Omega \\ & { = }f_{AS} (\omega )\int_{0}^{\infty } {A(\Omega )} \chi^{(3)} (\Omega ) \cdot (f_{pump} (\omega - \Omega ) \cdot E_{pump} (\omega - \Omega ))d\Omega \\ \end{aligned}$$

where

2 $$A(\Omega ) = \int_{0}^{\infty } {f_{pump} (\omega + \Omega )} E_{pump} (\omega + \Omega ) \cdot (f_{Stokes} (\omega ) \cdot E_{Stokes} (\omega ))^{*} d\omega$$

where $f$ represents the localized field response factor by the plasmonic structure, $\Omega$ represents the Raman energy level, ${\upchi }^{(3)}$ is the third-order susceptibility of detected sample, and ${\text{E}}_{pump}$ and ${\text{E}}_{Stokes}$ are exciting pump($\omega + \Omega$) and Stokes($\omega$) fields. It should be noted that different from the positive real number EF for single-frequency analysis, $f$ contains the imaginary part as the phase-modulation by the structure. From the Eq. (1), the spectral bandwidth and shape of the exciting light determines the final CARS spectrum. A better enhancement effect can be obtained by the larger half-height width of the excitation light spectrum. The interaction process of class convolution further widens the effective enhancement bandwidth. We define the integral of the CARS spectral intensity over the wavenumber axis as the enhancement factor for broadband CARS, considering both enhancement and spectral bandwidth, i.e., $G_{{{\text{broadband}}}} = \frac{{\int {|E_{AS} (\omega )|^{2} d} \omega }}{{\int {|E_{{_{AS} }}^{^{\prime}} (\omega )|^{2} d} \omega }}$, where $E_{AS}^{\prime } (\omega ) \propto \int_{0}^{\infty } {E_{pump} (\omega - \Omega )\chi^{(3)} (\Omega )} d\Omega \int_{0}^{\infty } {E_{pump} (\omega + \Omega ) \cdot } E_{Stokes}^{*} (\omega )d\omega \,$.

## Plasmonic structure description and analysis

**Figure 1:**
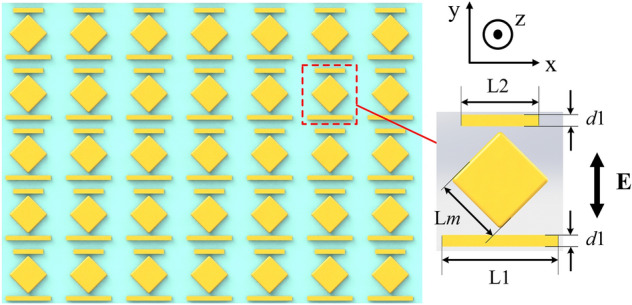
The diagram of the period Fano structure. The individual unit is shown in the right.

In this work, a new periodic plasmonic nano-structure is proposed for broadband CARS enhancement. The plasmonic nanostructure system with three units is as in Fig. 1. In order for better localized electric field, a square-shaped gold structure is set in the middle. For Fano-resonance process, asymmetrical parallel gold rods are set on both sides. The lower rod length is defined as L1 while the upper rod as L2. The side length of the middle structure is set as L_m_ = 180 nm. The periodicity of the structure is set as 400 nm. The distance between the two rods is 280 nm. The width of the two rods is d1 = 25 nm and the height of the whole structure is 30 nm. The polarization direction of the incident electric field is also shown in Fig. 1. The incident direction is along the z axis.

**Figure 2:**
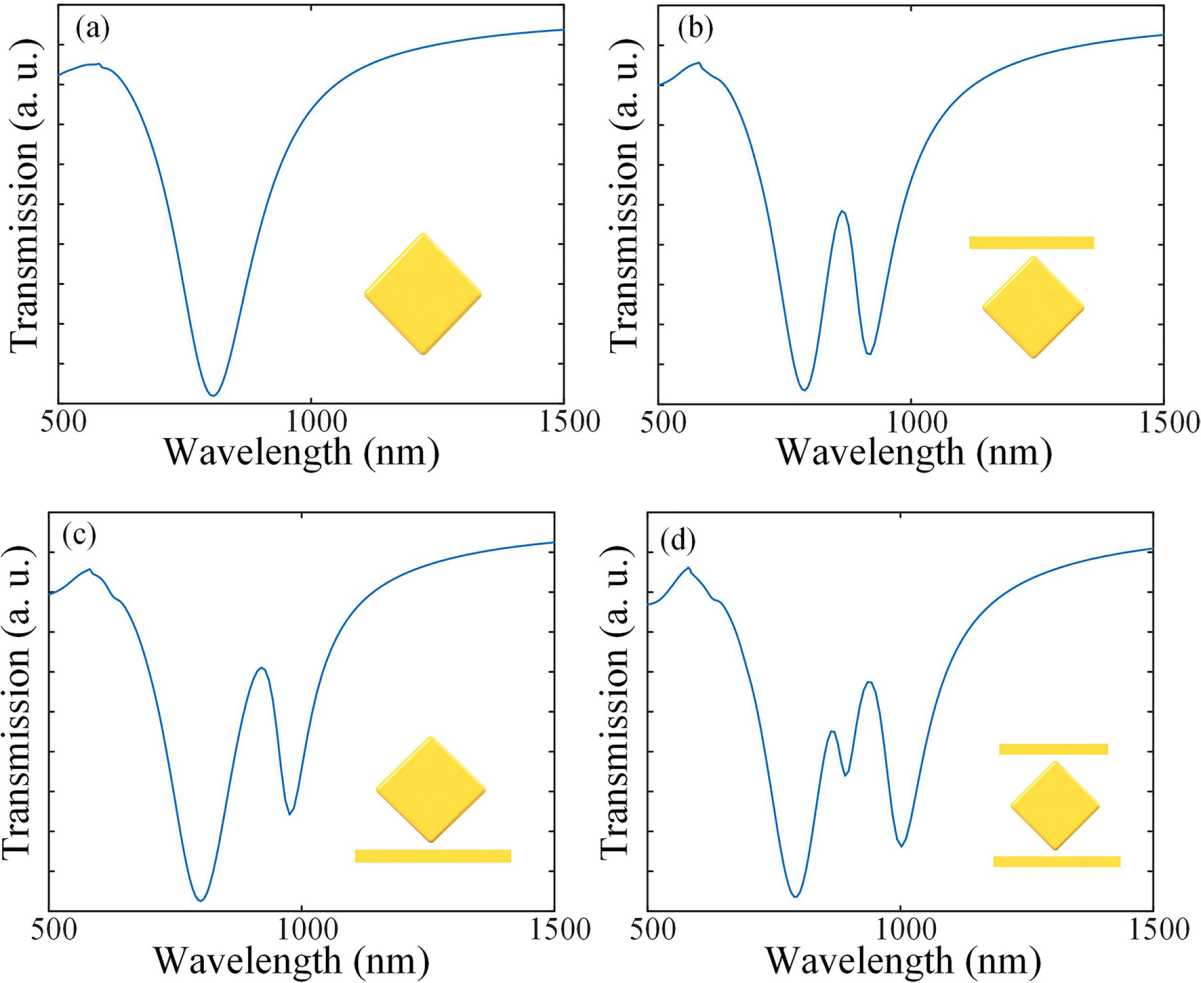
The transmission spectrum of the 4 types of the plasmonic structure. (**a**) Only the middle structure. (**b**) The middle structure and the top rod (L2 = 300 nm). (**c**) The middle structure and the rod below (L1 = 350 nm). (**d**) Complete three-part structure (L1 = 350 nm, L2 = 300 nm).

The transmission spectrum and electric field intensity distribution of the structure are calculated by a commercial software (FDTD solution). As Fig. 2d shows, two distinct Fano peaks are clearly observed when L1 = 350 nm and L2 = 300 nm. Like many typical metallic arrays with Fano resonance, the bright mode is provided by a broad dipolar resonance from the middle squared-shape component. From Fig. 2a, the transmission curve of the middle part shows a smooth and broadband lineshape providing the continuum for the narrow Fano resonance. While the narrow dark mode is generated from the coupling of the rod and the middle part. The upper and lower rods with different lengths contribute to the Fano peaks at different wavelengths, as shown in Fig. 2b,c. Hence, the final structure containing three main components shows a typical double Fano-resonance lineshape generated from the destructive interference of the bright and dark modes as in Fig. 2d.

**Figure 3:**
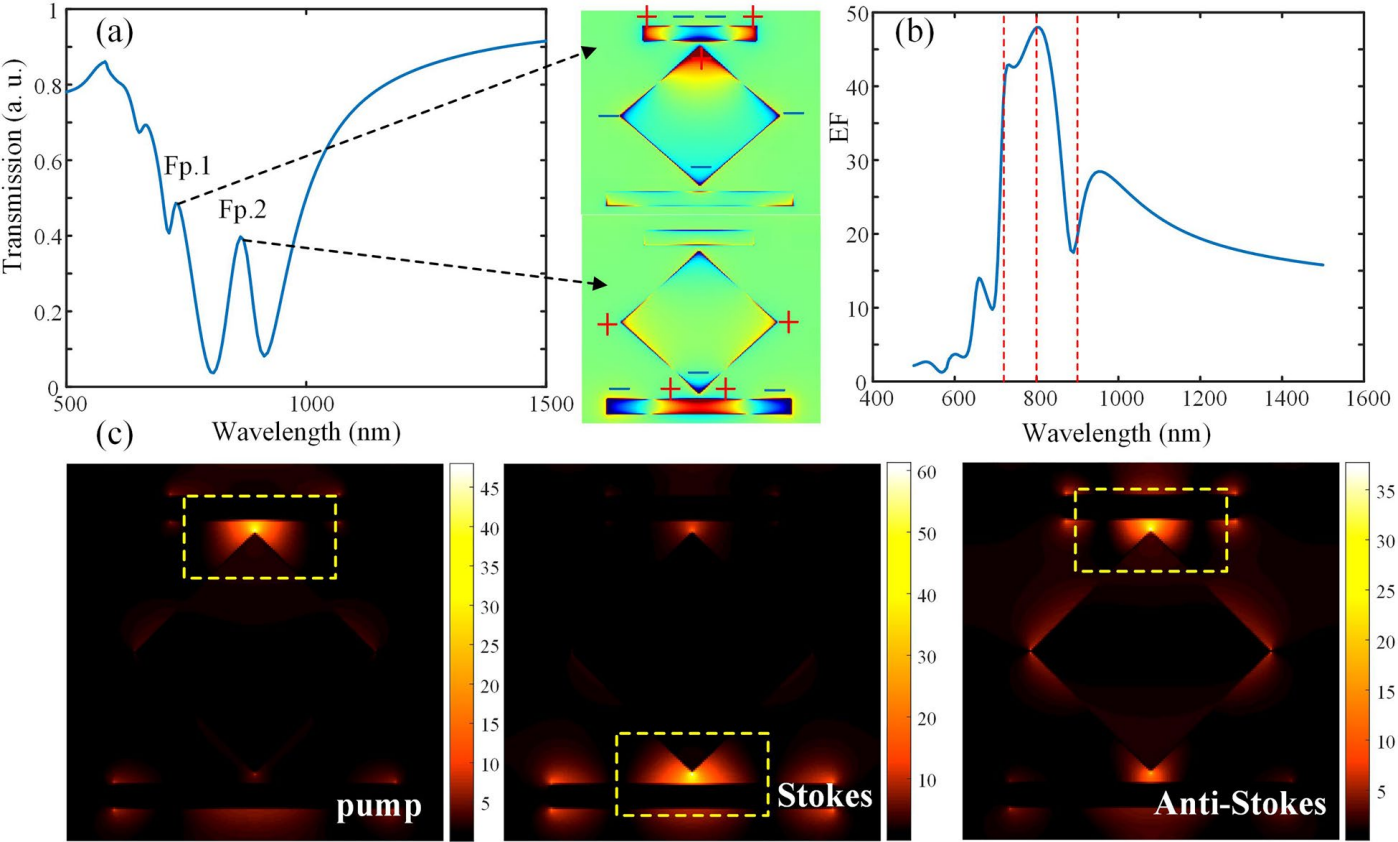
(**a**) The transmission spectrum of the structure with L1 = 300 nm and L2 = 180 nm. The two Fano peaks are marked as Fp.1 and Fp.2, which corresponds to the surface charge distribution at the two Fano peaks (723 nm and 870 nm) in the right. (**b**) The EF of different wavelengths at the maximum enhancement site. The red dotted lines are corresponding to 720 nm, 800 nm and 900 nm. (**c**) The electric field intensity distribution of the structure with L1 = 300 nm and L2 = 180 nm under the wavelengths of 800 nm, 900 nm and 720 nm.

To further understand the double Fano resonance, the surface charge distribution at the two Fano peaks are shown in Fig. 3a. The surface charge distributions at the two Fano peaks are also shown. The quadrupole plasmon resonance is visible on the two rods while the dipole plasmon resonance dominates on the middle part. Due to the strong coupling between the lower rod and the tip of the middle part, there is part of the opposite charge distribution at the tip, while the middle part shows a dipole charge distribution as a whole. The destructive interference between the narrow quadrupole and broad dipole resonances results in a double Fano resonances. The strong coupling effect of the resonance leads to the enhancement of the localized field. From the simulation results, the field enhancement factors for different wavelengths and spatial positions can be calculated. For the pump light of 800 nm, Stokes light of 900 nm and anti-Stokes light of 720 nm, the maximum single-frequency $G_{{{\text{SECARS}}}}$ can be up to 12 orders of magnitude ($G_{{{\text{SECARS}}}} = g_{{_{{\text{P}}} }}^{4} g_{{_{{\text{S}}} }}^{2} g_{{_{{{\text{AS}}}} }}^{2}$), when L1 = 300 nm and L2 = 180 nm after modifying the geometry. The EF of different wavelengths at the maximum enhancement site is shown in Fig. 3b. Obviously, the maximum enhancement site is in the interval region between the sharp point of the middle part and the rod due to the lightning rod effect, as shown in the yellow dashed box in Fig. 3c. The “hot spots” for pump and anti-Stokes wavelengths are corresponding to the Fano resonance generated from the coupling between the upper rod and the middle part, while the “hot spots” for Stokes wavelength is corresponding to the Fano resonance generated from the coupling between the lower rod and the middle part. In addition, sharp tips are unfavorable for fabricating. Hence, considering the realistic fabrication condition, we also simulate the chamfered middle squared-shape part with different rounded corner radius, and the moderate chamfered degree doesn’t affect much to the $G_{{{\text{SECARS}}}}$.

**Figure 4:**
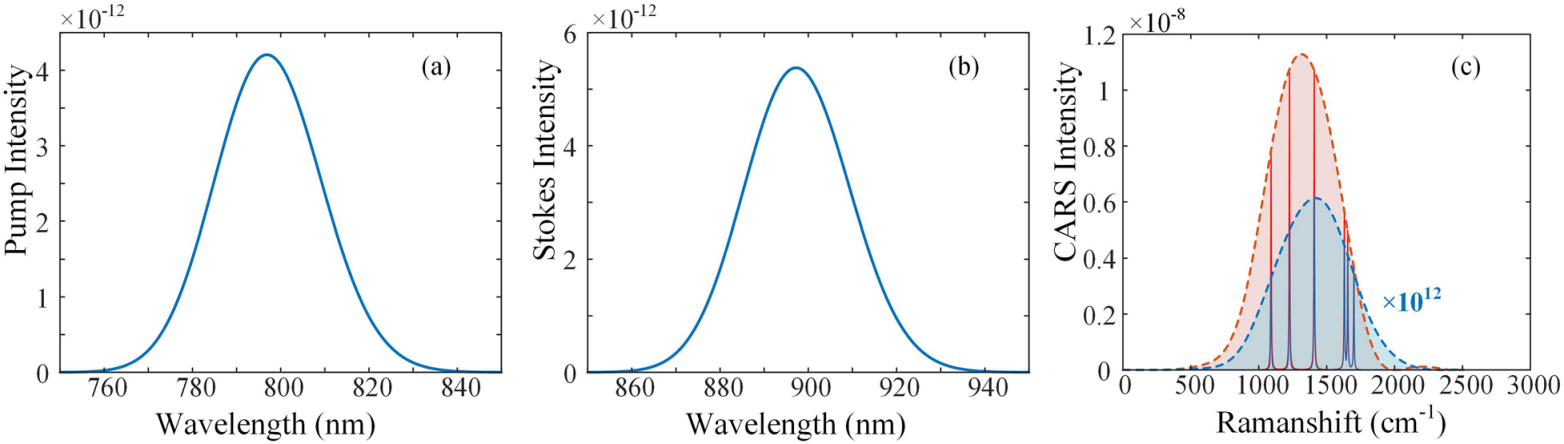
The simulation spectra of the pump (**a**), Stokes (**b**) and CARS (**c**). The light red and light blue regions in (**c**) correspond to the enhanced and unenhanced CARS spectra, respectively, where the intensity of the unenhanced CARS spectrum is amplified by 12 orders of magnitude.

Due to the Fano peaks cover a wide range of wavelength axis, the structure shows huge potential in broadband CARS enhancement. In fact the final broadband CARS spectrum depends on the different excitation methods, and we simulate the Fourier-transform CARS (FT-CARS) excitation process as an example. FT-CARS uses two replicas of pulses with changing relative delay to retrieve the molecular vibrational response. With the spectrum and delay of the excitation pulses as the input parameters, the intensity of anti-Stokes scattering can be calculated using the excitation equations of CARS as Eq. (1). The anti-Stokes intensity is calculated for each relative delay, and the retrieved intensity of different delay, which is identical to the molecular vibrational response, is Fourier transformed to obtain the final Raman spectrum. More detailed simulation process of FT-CARS can be referred to^20^.The intensity of the pump and Stokes is assumed to be Gaussian-shaped distribution to the frequency, whose spectra are shown in Fig. 4a,b. The wavelength ranges of pump light and Stokes light are 750–850 nm and 850–950 nm, respectively. For the structure with L1 = 300 nm and L2 = 180 nm, the anti-Stokes intensity with the plasmonic structure is enhanced by ~ 12 orders of magnitude compared to that without the corresponding structure in the Raman region of 500–2000 cm^−1^. We select several characteristic Raman peaks in the fingerprint region: the C–C bond at 1090 cm^−1^, the C=C stretching vibration at 1655 cm^−1^, the symmetric CO_2_-stretching vibration at 1408 cm^−1^, the asymmetric CO_2_-stretching vibration at 1700 cm^−1^, the C=O stretching vibration at 1630 cm^−1^, the aromatic vibration at 1226 cm^−1^, and the aromatic vibration at 1226 cm^−1^. It can be found in Fig. 4c that the intensity of all these characteristic peaks is enhanced by ~ 12 orders of magnitude under the resonance effect. This verifies that the designed structure has a good enhancement effect in a wide range of wavenumbers, and the broadband enhancement factor $G_{{{\text{broadband}}}}$ is 1.73 × 10^12^, which is consistent with that of a single-frequency SECARS in magnitude.

**Figure 5:**
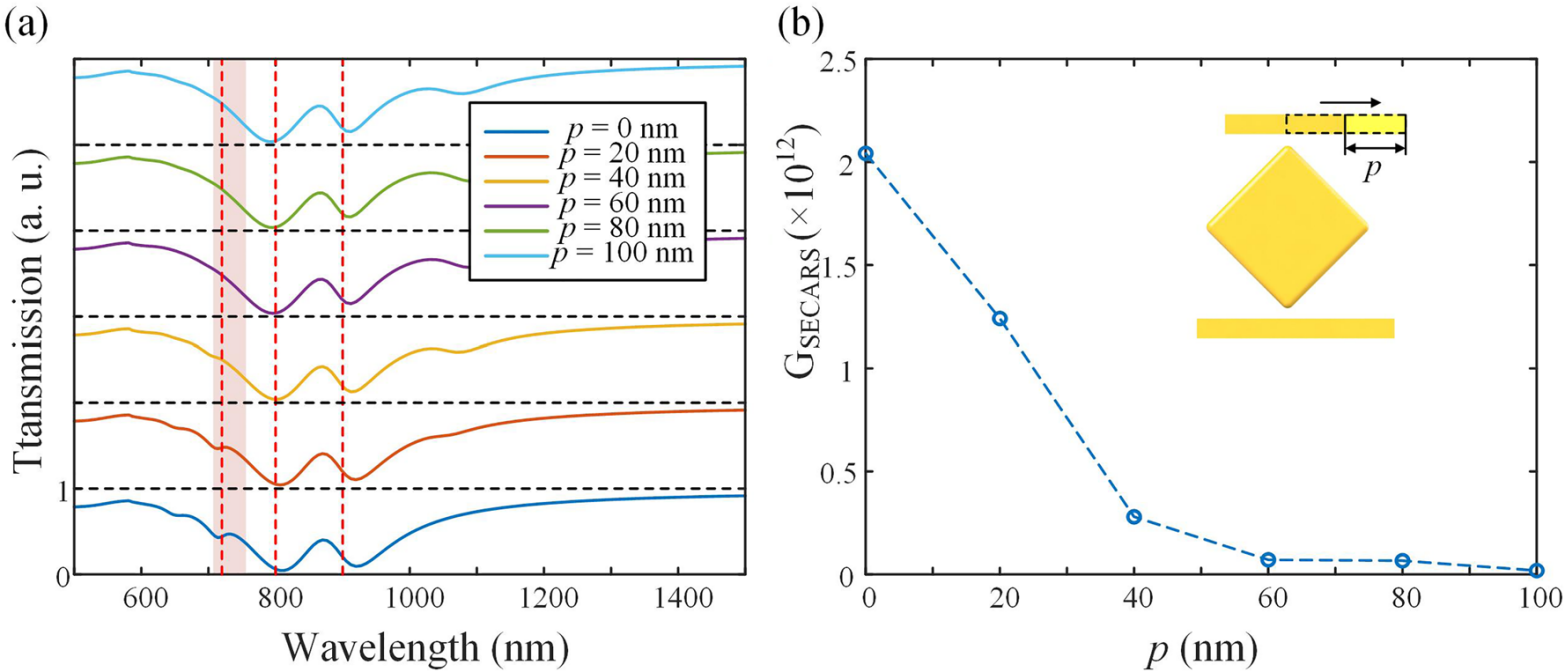
The transmission spectra (**a**) and $G_{{{\text{SECARS}}}}$ (**b**) of the structure of L1 = 300 nm and L2 = 180 nm with different L2 positions. In (**a**), the red region is the position of one of the Fano peaks from the coupling between L2 and the middle part. The red dashed lines are corresponding to 720 nm, 800 nm and 900 nm.

By changing the geometric characteristics of the plasmonic structure, the Fano-resonance properties are modulated for the application of SECARS. First, the position of the shorter rod is changed for the structure. It should be noted that the middle transmission dip comes from the middle part of the plasmonic structure, and the double Fano peaks are on both sides. The coupling between shorter rod and the middle part contributes to the Fano peak position at around 700 nm as shown in Fig. 5a. It can be found that the double Fano peak converts to a single Fano peak as the short rod position moves to the edge. The near-field coupling between the middle part and the short rod is weaken due to the spatial location of the two components away from each other. At the same time, the total $G_{{{\text{SECARS}}}}$ of single-frequency SECARS decreases a lot as the Fano resonance weakens, as shown in Fig. 5b. This also confirms that Fano resonance has a significant impact on local field enhancement for SECARS in our plasmonic structure. From the above simulation results, the position of the two rods is more suitable in the middle for larger EF, thus in the subsequent simulation, the *p* is set to 0.

**Figure 6:**
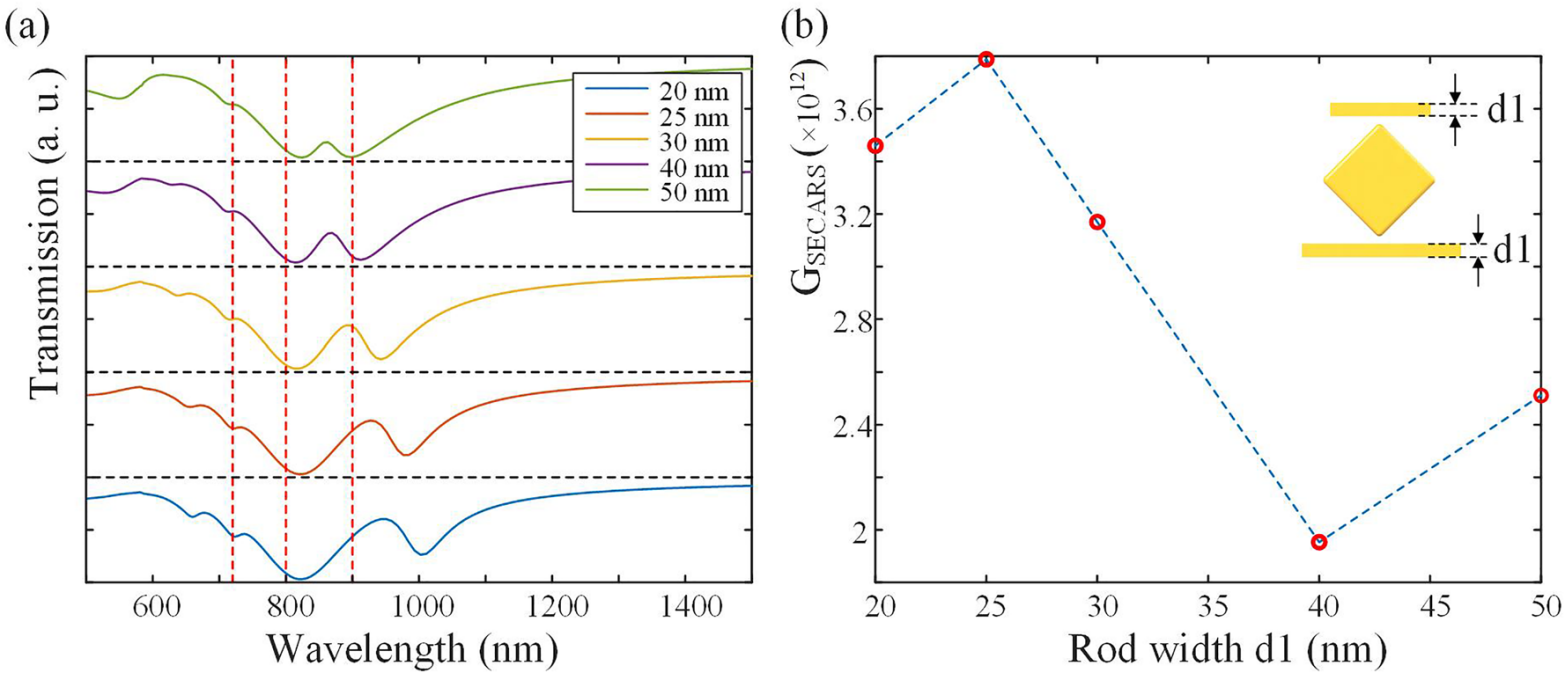
The transmission spectra (**a**) and $G_{{{\text{SECARS}}}}$ (**b**) of the structure of L1 = 300 nm and L2 = 180 nm with different rod widths d1. The red dashed lines are corresponding to 720 nm, 800 nm and 900 nm.

The rod width d1 is also changed for a larger $G_{{{\text{SECARS}}}}$. As shown in Fig. 6a, it can be found that the rod width mainly affects the position of the right Fano peak. The right Fano peak comes from the coupling of the longer rod to the middle structure, which indicates that the effect on Fano resonance of the rod width is more significant under a longer rod length. As in Fig. 6b, with d1 = 25 nm, the largest $G_{{{\text{SECARS}}}}$ is obtained, and the rod width is set to 25 nm in the subsequent simulation.

Further, another critical geometric factor, the length of the two rods, is also varied for controlling the Fano resonance for better SECARS. We simulate the different geometric structures by varying the length of the upper rod under fixed L1(300 nm, 325 nm, 350 nm). There is a red shift of the right Fano peak when the length of L2 is fixed while increasing L1. Similarly, for fixed L1 with increasing L2, there is also a red shift of the left Fano peak. When L2 is relatively small, the left Fano peak is weak and in the shorter wavelength position, which is not easy to observe. When L2 reaches a certain length, three peaks occur in the transmission spectrum. Then continue to increase L2 length, the left Fano peak crosses the middle absorption peak generated from the middle structure and gradually approaches the right Fano peak. From above simulation results, the length of the rod directly affects the Fano peak position and intensity simultaneously, therefore by changing the length of L1 or L2, the absorption peak position can be flexibly modified for larger $G_{{{\text{SECARS}}}}$.

**Figure 7:**
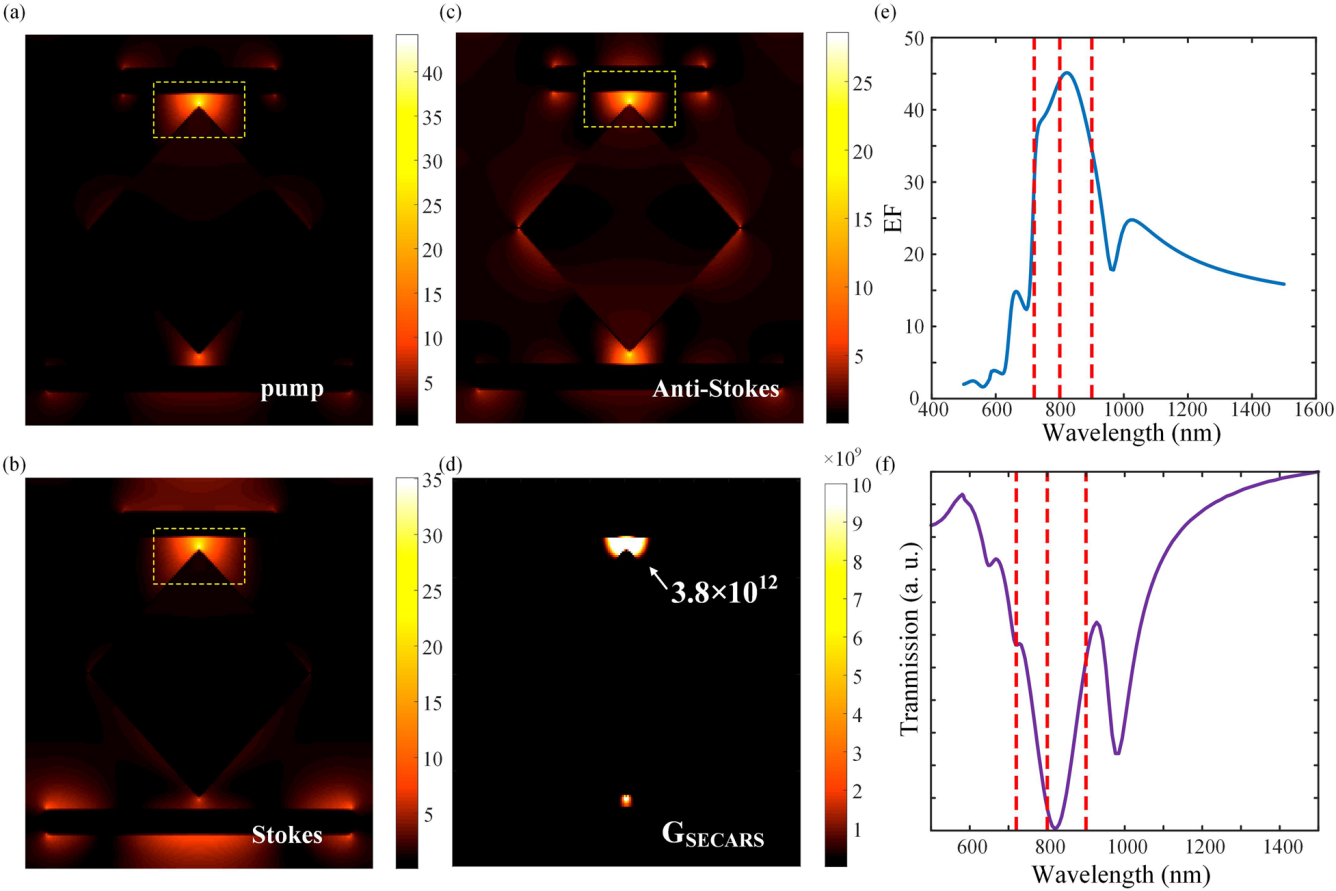
The electric field distribution at pump, Stokes and anti-Stokes wavelength (**a**–**c**) and the $G_{{{\text{SECARS}}}}$ (**d**) of the structure with L1 = 350 and L2 = 175 nm. (**e**) The EF of different wavelengths at the maximum enhancement site of the structure with L1 = 350 and L2 = 175 nm. The red lines are corresponding to 720 nm, 800 nm and 900 nm. (**f**) The transmission spectrum of the structure with L1 = 350 nm and L2 = 175 nm.

We first consider the single-frequency SECARS and calculate $G_{{{\text{SECARS}}}}$ under varying L2 lengths from 100 to 250 nm with fixed L1(300 nm, 325 nm, 350 nm). Actually, 12 times magnitude of $G_{{{\text{SECARS}}}}$ always appear in the condition that the double Fano peaks set around 700–900 nm. Like for L1 = 300 nm and L2 = 180 nm, the Stokes light wavelength sets at the right Fano dip, the pump light wavelength at the middle dip between the two Fano peaks, and the anti-Stokes light wavelength at the left Fano peak, the relatively large $G_{{{\text{SECARS}}}}$ of 2.7 × 10^12^ is obtained. It can be found that the intense “hot spot” region always sets at the gap region where strong coupling between the two components of the plasmonic structure exists. According to Ouyang’s work^21^, it is verified that the enhancement in the pump and anti-Stokes beams plays a more critical role in the overall enhancement than that in the Stokes beam, and thus even for the dominate “hot spot” region of the Stokes light wavelength locates at the different position (in the lower gap region) with that of pump and anti-Stokes light, there is still a localized field enhancement effect in the upper gap region and thus the total $G_{{{\text{SECARS}}}}$ is still large in the upper “hot spot” region. For the condition of L1 = 300 nm and L2 = 180 nm, the effective enhancement region is calculated and $G_{{{\text{SECARS}}}}$ of around 0.33% of the periodic structure unit area is over 10^8^. We assume that the excitation light spot diameter is 1 μm, and the “hot spot” region with $G_{{{\text{SECARS}}}}$ over 10^10^ is more than 750 nm^2^ size which is large enough for single-molecular monitoring^5^. By further modulating the rod length, the same “hot spot” positions of the three beams can be achieved. Particularly, For L1 = 350 and L2 = 175 nm, both the pump and Stokes wavelength locate in the middle dip between the two Fano peaks. The enhancement of pump and Stokes light comes from the resonant absorption of the middle structure, and the enhancement of anti-Stokes light comes from the Fano resonance. Under the condition, the “hot spots” of the three wavelengths are at the same place as shown in Fig. 7a–d. As shown in Fig. 7e, the EF of pump, Stokes and anti-Stokes light are larger than 30 and the final $G_{{{\text{SECARS}}}}$ reaches the largest 3.8 × 10^12^. The transmission spectrum is also shown in Fig. 7f.

**Figure 8:**
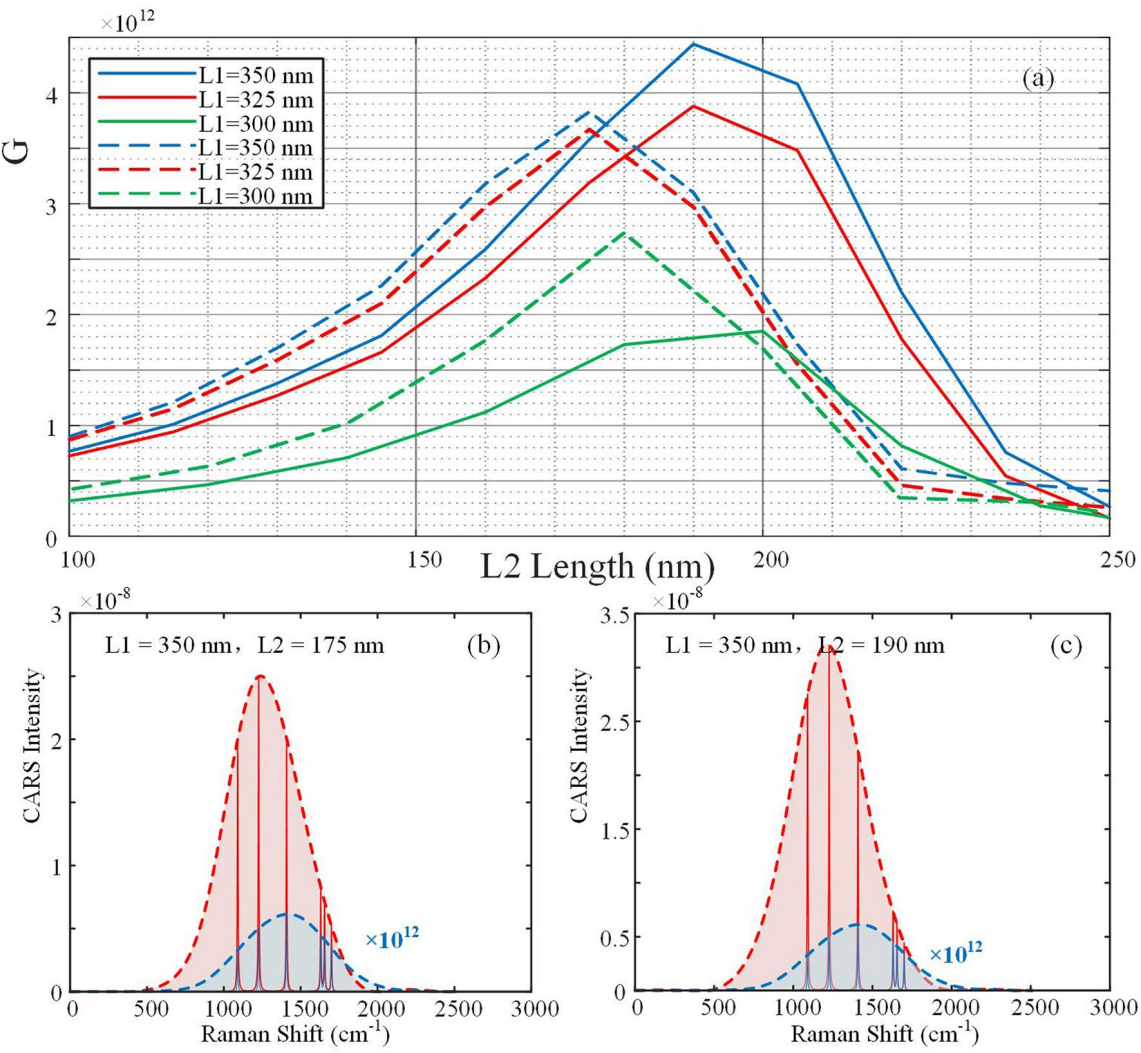
(**a**) The $G_{{{\text{broadband}}}}$(solid) and single-frequency $G_{{{\text{SECARS}}}}$(dotted line) under different L1 and L2 lengths. (**b**–**c**) The simulation CARS spectra with L1 = 350 nm and L2 = 175 nm (**b**) and L1 = 350 nm and L2 = 190 nm (**c**). The light red and light blue regions correspond to the enhanced and unenhanced CARS spectra, respectively, where the intensity of the unenhanced CARS spectrum is amplified by 12 orders of magnitude. Some characteristic Raman peaks are also presented.

For broadband SECARS, we also analysis the $G_{{{\text{broadband}}}}$ of the structures under different L1 and L2, and the results are shown in Fig. 8a (The broadband light source settings are the same as above). The structure has a high enhancement effect over a wide range of wavenumbers for different geometric configurations. To better compare the two evaluation factors, we represent the single-frequency $G_{{{\text{SECARS}}}}$ in Fig. 8a as well. It can be found that for both the single-frequency and broadband configurations, under a fixed length of L1, the EF increases to a certain maximum and then gradually decreases with the increase of L2 length, keeping a consistent trend of change. The difference is that the geometric configuration corresponding to the maximum EF is not the same, and a better broadband enhancement effect can be realized at a larger L2 length. For instance, for L1 = 350 nm and L2 = 175 nm, the structure shows largest $G_{{{\text{SECARS}}}}$ at the strongest electric field intensity position, while it shows smaller $G_{{{\text{broadband}}}}$ compared with the condition of L1 = 350 nm and L2 = 190 nm. The broadband enhancement effect can be verified from the CARS spectra in Fig. 8b,c. The structure with L1 = 350 nm and L2 = 190 nm has larger CARS intensities over a wider range of wavenumbers and therefore exhibits higher EF. Simulation results demonstrates that the evaluation of single-frequency EF is not applicable to broadband SECARS systems. Therefore, for the case of broadband SECARS, the evaluation factor of single-frequency SECARS is not sufficient to obtain the optimal geometric configuration. The excitation properties of broadband CARS need to be considered, and the broadband EF has a more comprehensive evaluation and can be used to guide the design of more optimal enhanced micro-nano structures under broadband CARS system.

## Conclusion

In this work, we have demonstrated that the specific plasmonic structure can obtain up to ~ 12 orders of magnitude electric field intensity enhancement. And the two Fano peak positions can be independently tuned by changing the geometrical structure parameters. Based on the broadband CARS principle, the evaluation methodology of the enhancement factor is proposed and the final anti-Stokes bandwidth and intensity depends on the exciting light bandwidth and enhancement factor. It is verified that this special structure has a ~ 12 orders of magnitude of enhancement for the wavenumber region of 500–2000 cm^−1^. This broadband property of the plasmonic structure helps to the broadband CARS configuration, such as Fourier-transform CARS and spectral-focusing CARS, and it will be a useful tool for increasing the signal-to-noise ratio especially for highly sensitive biochemical detection^16^.

## Data Availability

The simulation data used and analysed during the current study are available from the corresponding author on reasonable request.
